# Improving the study of error monitoring with consideration of behavioral performance measures

**DOI:** 10.3389/fnhum.2014.00178

**Published:** 2014-03-26

**Authors:** Hans S. Schroder, Jason S. Moser

**Affiliations:** Department of Psychology, Michigan State UniversityEast Lansing, MI, USA

**Keywords:** error-monitoring, post-error adjustments, post-error slowing, post-error accuracy, error-related negativity, error-related brain activity, performance-monitoring

The field of error monitoring is one of the richest and fastest growing research areas within human neuroscience. In particular, applied researchers in clinical psychology and psychiatry are excited about the potential for this research to inform models and treatments of psychopathology. Neuroimaging techniques including event-related brain potential (ERP) and functional magnetic resonance imaging (fMRI) have highlighted the fascinating ways in which the brain detects and responds to errors and how these processes go awry in clinical populations. However, an increased focus on the brain activity itself has resulted in a neglect of behavioral performance, including the important adjustments that follow mistakes. Here, we suggest that the consideration of task performance and, in particular, post-error behavioral adjustments (PEBAs), substantially improves our understanding of the function of error-related brain activity. Critically, this entails controlling for behavioral differences and reporting multiple PEBAs, especially post-error accuracy. We urge researchers to consider the full range of behavior in order to foster a richer understanding of how individuals detect and bounce back from their mistakes.

## Overall performance

Most studies of error monitoring use simple reaction-time (RT) tasks, such as the Eriksen flanker task (Eriksen and Eriksen, 1974), to elicit errors while brain activity is recorded via EEG or fMRI. Although the focus of this neuroimaging research is naturally on the brain, accumulating evidence indicates that differences in how individuals respond in the task (e.g., RT or accuracy) influence brain activity tremendously (Hajcak et al., 2004; Yarkoni et al., 2009; Grinband et al., 2011; Carp et al., 2012). This is particularly pertinent for activity within the anterior cingulate cortex (ACC), a region of the medial frontal cortex (MFC) and the primary focus of error-monitoring research (Carter et al., 1998; Shackman et al., 2011; Ullsperger et al., 2014). Activation within the ACC, especially when time-locked to errors, is often inversely related to the actual number of errors committed (Gehring et al., 1993; Holroyd and Coles, 2002; Yeung et al., 2004), such that reduced ACC activity often coincides with increasing errors. This finding has been incorporated into theories relating the ACC to conflict monitoring (Botvinick et al., 2001), reinforcement learning (Holroyd and Coles, 2002), and action-outcome violations more broadly (Alexander and Brown, 2011), among many others.

However, the fact that ACC inversely scales with errors is important in all studies of error monitoring, for example, in clinical studies comparing brain responses to errors between a psychiatric sample and a healthy control sample. This point is relevant for two recent studies of the error-related negativity (ERN), an ERP index of early error monitoring processes localized to the ACC (Falkenstein et al., 1991; Gehring et al., 1993, 2012). One study found that individuals diagnosed with major depressive disorder (MDD) showed enlarged ERN compared to healthy controls (Tang et al., 2013), but also found that the MDD group responded significantly more slowly and committed fewer errors (*p* = 0.06). The authors acknowledged they could not disentangle the ERN findings from the likely influence of psychomotor retardation, a common symptom of depression in which responses are slower (and perhaps more careful). Another study found that individuals diagnosed with internet addiction disorder (IAD) showed reduced ERN compared to healthy controls (Zhou et al., 2013), but also found that the IAD group responded significantly faster and committed more errors. Neither of these studies ruled out the influence of behavior on the ERN findings. We believe that the true incremental value of utilizing neuroimaging technology is to reveal differences that cannot be observed with or explained by behavior alone. It is therefore important for studies that find overall behavioral differences to control for these differences in brain activity analyses through covariate analyses or by selecting subsamples of participants matched on behavior (e.g., Riesel et al., 2011; Bartholow et al., 2012).

When groups are matched for behavior, interpretations of error-related brain activity are more straightforward. Studies of anxiety and its disorders consistently demonstrate enhanced error-related ACC activity, despite unaffected performance (e.g., Ursu et al., 2003; Hajcak, 2012; Moser et al., 2013). We have suggested this “neurobehavioral signature” reflects compensatory effort by which anxious individuals require more resources to achieve comparable performance as non-anxious individuals (Moser et al., 2013, 2014). Note that increased ACC activity and superior performance might confer optimal functioning, whereas increased ACC and poorer performance would confer ineffective performance (e.g., Eysenck et al., 2007). Importantly, we believe error-monitoring brain activity and behavior (e.g., RT, accuracy) are linked; we are not suggesting that they are disconnected when brain differences emerge in the absence of behavioral differences or vice versa. Our main point here is that integrating both brain and behavioral data allows for more informative interpretations that consider multiple sources of available evidence.

## Post-error behavioral adjustments

Understanding how individuals adjust to their mistakes on subsequent trials is crucial for a comprehensive study of adaptive error monitoring. There are three types of PEBAs (reviewed by Danielmeier and Ullsperger, 2011). Post-error slowing (PES)—by far the most commonly reported PEBA—refers to the slowing of RT on trials that follow errors, relative to trials that follow correct responses (Rabbitt, 1966). The function of PES is a matter of debate (Danielmeier and Ullsperger, 2011; Dutilh et al., 2012); generally speaking, some suggest it reflects increased response caution to improve performance on subsequent trials (Botvinick et al., 2001), while others suggest it simply reflects an off-task orienting process to novel or infrequent events that is somewhat irrelevant to the task (Notebaert et al., 2009). Regardless of interpretation, it is clear that task-specific parameters (e.g., cognitive demand, length of intertrial interval, presence of error awareness ratings) greatly influence PES and its functional significance (Jentzsch and Dudschig, 2009; Grutzmann et al., 2014). Post-error accuracy (PEA) refers to the accuracy on trials following errors relative to trials following correct responses. Unlike the nearly ubiquitous slowing of RTs following errors (PES), accuracy is not always higher on trials following errors (Danielmeier and Ullsperger, 2011; Schroder et al., 2012). We contend that PEA is a more straightforward metric of post-error *adaptation* than PES (Moser and Schroder, 2012; Schroder and Infantolino, 2013), because accuracy is almost always desired (as opposed to merely slower responses, as in PES) in seemingly any task context. The final and least studied PEBA is post-error reduction of interference (PERI), which refers to the reduction in interference-related RT effects following errors. PERI has only been examined in a handful of studies and is not yet well understood (Danielmeier and Ullsperger, 2011).

Although PES is commonly assumed to be adaptive in all contexts, it is clear from accumulating evidence that this is not always the case (e.g., Gehring et al., 2012). For instance, PES is typically not or negatively correlated with PEA, and these two adjustments are likely mediated by dissociable neural mechanisms (Carp and Compton, 2009; Danielmeier et al., 2011). Although we acknowledge the functional significance of PES depends on many factors, we argue that it is difficult to determine whether or not it is “adaptive” without examining other behavioral markers of adaptation such as PEA. In a recent rodent study, Narayanan et al. (2013) investigated the effects of MFC inactivation on “adaptive control” in terms of both neural activity and behavioral adjustments following errors in a time-estimation task. They found reduced low-theta oscillations and reduced PES among rodents in the inactivation condition, suggesting MFC is necessary for adaptive control. They also suggested their rodent model and task was appropriate for understanding the effects of brain stimulation and psychopharmacological agents. However, PEA was not examined in the rodent or the comparison human sample, raising questions about the utility of PES in that study.

A closer look at the clinical literature reveals a highly inconsistent relationship between PES and various types of psychopathology including attention deficit hyperactivity disorder (ADHD), obsessive-compulsive disorder (OCD), and schizophrenia. For instance, in some studies, PES was modulated among individuals with symptoms of ADHD (Krusch et al., 1996; Sergeant and van der Meere, 1988; Schachar et al., 2004; Wiersema et al., 2005; Yordanova et al., 2011; Shiels et al., 2013), but in other studies, ADHD symptoms were not associated with PES (Jonkman et al., 2007; Van Meel et al., 2007; Herrmann et al., 2009; Van De Voorde et al., 2010). A great variety of tasks were used across these studies (in fact, no two studies used the same exact task and parameters), which likely contributes to the mixed findings (cf. Schroder et al., 2013). For tasks in which the subjects are aware of the trial sequence following errors (e.g., knowing that errors on lure trials will be followed by a subsequent lure stimulus in the next few trials, Hester et al., 2007), PES may be a necessary strategy to slow down and recover performance in anticipation. On the other hand, tasks in which trial sequences are presented randomly (e.g., the flanker task; which likely make up the majority of error-monitoring studies), the utility of PES becomes much less clear. Nonetheless, very few error-monitoring studies seriously consider the task-specific contexts in which PES does or does not occur, and whether or not it is objectively adaptive in a given context (e.g., if it coincides with or is related to PEA). At the very least, the lack of consistency across the above-mentioned studies speaks against the “universally” adaptive nature of PES. We therefore urge researchers not to assume PES is adaptive unless they have examined and reported data that support its utility.

## Brain-behavior correlations

Finally, brain-behavior correlations provide information about the *functional* significance of error-related brain activity. Predominant theories suggest that error-related ACC activity signals for adaptive adjustments in performance such as PES or PEA (e.g., Botvinick et al., 2001; Holroyd and Coles, 2002; Yeung et al., 2004). The extent to which error-related brain activity is associated with PEBAs, however, is not well understood (Danielmeier and Ullsperger, 2011), and we contend that this is largely due to a lack of reporting correlations between brain activity and PEBAs. Reporting of such relationships will allow for a clearer understanding in this regard.

Studies of clinical disorders may especially benefit from examining brain-behavior correlations. Cavanagh et al. (2011) found that individuals with depression showed hyperactive error signals, but also that these hyperactive error signals were tightly coupled with accurate avoidance learning behavior; this coupling was not present in the non-depressed group. Other brain-behavior correlation differences between depressed and non-depressed groups (Compton et al., 2008; Holmes and Pizzagalli, 2008; Schroder et al., 2013) suggest depression may be associated with a failure to recruit adaptive resources in order to recover from mistakes. Similar analyses have revealed that diminished ACC responses to errors are associated with poorer error awareness among chronic cannabis-using individuals (Hester et al., 2009). Correlations between error-related brain activity and behavior outside the task context provide further insight into the real-world functional significance of these neural phenomena (e.g., Foti et al., 2012, Marhe et al., 2013; Moeller et al., 2014).

## Conclusion

Here, we sought to point out the value and utility of considering *behavior* and neural activity together in an integrated framework of error monitoring. Our overall message is that incorporating behavioral performance measures (RT *and* accuracy)—especially following errors—is necessary for optimally informative interpretations regarding the “adaptive” nature of brain activity elicited by errors. When interpreting between-group comparisons of brain activity, it is necessary to consider any behavioral differences between the groups. Researchers can substantially expand our comprehension of post-error adjustments by reporting all three PEBAs (PES, PEA, and PERI), the correlations between them, and brain-behavior relationships between brain activity and these adjustments. Although here we have focused on immediate adjustments on the very next post-error trial, future research could also explore how adjustments on immediate post-error (*n* + 1) trials influence brain activity and performance several trials after the initial error (cf. Hester et al., 2007). Overall, delineating the interplay between brain and behavior will considerably improve our understanding of how individuals detect and learn from their mistakes. Moreover, this fuller picture will more accurately inform what it means when these processes go awry in psychopathology and, ultimately, how to correct them (e.g., Sylvester et al., 2012).
